# Supplementary material for: Role and modulation of various spinal pathways for human upper limb control in different gravity conditions
Source: PLoS Comput Biol. 2025 Jan 6;21(1):e1012069. doi: 10.1371/journal.pcbi.1012069 (PMC11737853; doi:10.1371/journal.pcbi.1012069)
Supplement: Attachment — Submitted filename: PLoS CB review.pdf [file pcbi.1012069.s001.pdf]

The authors would like to sincerely thank the reviewer for his.her time spent on reviewing this work and insightful comments, which have significantly enhanced the manuscript. We have carefully considered his.her feedback and addressed it in the revised version. Below, we respond to each of the reviewer's questions and comments in detail. All modifications in the revised manuscript are highlighted in blue for easy reference.

Reviewer #1:

How the brain and spinal cord (SC) work together to implement motor adaptation, is an important question. Therefore I was quite interested to read this paper, and the work reported has been carried out with sufficient care. However, having read the paper, I am not sure what fundamental techniques or physiological principles I have learned from it. Using a neuromechanical model considering a Hill model of muscle-tendon mechanics and various reflexes including the monosynaptic stretch reflex and heteronymous reflexes, the paper (a) first evaluates which reflexes have most relevant effect in a single joint elbow movement up and down. It then (b) examines how the gravity involved in these movements can be learned by either the brain, i.e. through identification of a simple motor command's parameters, or through identification of the parameters of two most relevant reflexes. Part (a) is presented well, though it could have a systematic sensitivity analysis, that could be an interesting contribution. My problem is more with part (b) as I do not understand what is the contribution here. As both the brain and SC participate to the control and their parameters are combined, it is not surprising that either can be used to adapt the behaviour. In fact from an identification point of view one could consider the combined parameters and use techniques such as observability to understand what each can contribute to or whether some adaptation could not be provided by one of them. Also as only one task is considered it is difficult to make any conclusion about whether adaptation of the spinal reflexes would be sufficient to provide motor adaptation in general.

Answer: We would like to thank the reviewer for his.her valuable feedback. Regarding part (a) we fully agree on the added value of sensitivity analyses and we integrated them in the reviewed version. The resulting first-order indices confirmed that stretch reflexes have the stronger effect on the root mean squared error (RMSE), smoothness, and deviation due to perturbation. We would also like to point out here that the trajectory is indeed an elbow flexion extension, but with the shoulder flexion-extension also unrestricted, so that we consider a double joint control.

Regarding part (b), both the brain and the SC indeed participate in the control. Nevertheless, we would like to highlight that the SC stretch reflexes tend to bring the musculoskeletal system to its resting position (both physiologically and by definition in the model). Thus it is not that straightforward that modulation of SC reflexes allows for gravity compensation (i.e. reproduction of a target double joint trajectory in different gravitational environments). We agree that our study is limited by the single simulated task. We chose to favour the analysis of many control scenarios on one task rather than less control scenarios on more tasks. To improve this point, we run our different scenario pipelines on another circular trajectory and observed similar trends. We added these new results and tried to clarify our main messages in the introduction and in the discussion in the reviewed version.

Another problem that I see is that (as mentioned in the text) some of the same authors just published a paper in this journal, with a cerebellar model and a simple SC model. I have looked at that paper and like it and see a clear message and contribution. In contrast, I have problem to identify a clear message or contribution in the present manuscript.

Answer: First we would like to express our gratitude for your interest and positive feedback on our previous paper regarding the interaction between cerebellar learning and spinal circuits for upper limb motor control. While this paper highlights the role of simple spinal circuits (stretch reflex + reciprocal inhibition) in facilitating complex cerebellar learning, the current paper focuses on the role and modulation of numerous spinal circuits in handling perturbation and gravity compensation, in interaction with simple brain commands. The previous paper also demonstrated the role of stretch reflexes in handling perturbations, but the current paper explores a wide range of spinal circuits and their modulation in various scenarios. While spinal reflex modulation was not addressed in the earlier work, it was suggested as a topic for further investigation in the discussion. In this paper, we have begun to integrate spinal modulation modeling with simple brain commands to enhance interpretability, as you noted that it can be challenging to identify individual contributions when multiple components are involved in control. We tried to clarify this point in the introduction and discussion.

Some technical issues that are on the muscle model. The monotonic stiffness increase with activation/force is not considered, which is one of the most important characteristic of muscle mechanics, see e.g. Kirsch et al. (1994), IEEE Transactions on Biomedical Engineering. Also the Hill muscle model considers both the force length and force velocity relationships. However, is it important or valid here to consider the force-length relationship, as how it influences the force depends on the muscle and movement, see e.g. Murray et al. (2000), Journal of Biomechanics.

Answer: We would like to thank the reviewer for his technical observations. We believe that we do consider these muscle characteristics by using Hill-based Millard muscle model, see e.g. Millard et al. (2013) Journal of Biomechanical Engineering, at least in quasi-static consideration. This model describes the muscle force as follow:

$F(a, l, v) = a * f_l(l) * f_v(v) + f_p(l)$

with a the muscle activation,
l the muscle length,
v the muscle velocity,
f_l the force-length relationship,
f_v the force-velocity relationship,
f_p the passive force-length relationship.

On one hand, the monotonic stiffness increase with activation/force is implemented through the factor $a * f_l(l)$. The stiffness yield and steady-state reported by Kirsch et al. (1994) under

step perturbation and stochastic perturbation are also integrated in the force-length relationship.
On the other hand, the operating ranges of arm muscles emphasised by Murray et al. (2013) are also reflected in the simulation. Each modeled muscle encompasses a specific range of length variation depending on its path across the joints. The relevant segment of the force-length curve is then applied as described in Murray et al. (2013).

Understanding how the brain and spinal cord (SC) collaborate to achieve motor adaptation is a significant question in neuroscience. I was therefore intrigued to read this paper on this topic. However, after reading it, I am uncertain about the fundamental techniques or physiological principles that I have gained from it.

Answer: We thank you again for your interest and valuable review, and we hope that our answers to your comments clarified our study.
